# Best practices in the laboratory diagnosis, prognostication, prediction, and monitoring of Graves’ disease: role of TRAbs

**DOI:** 10.1186/s12902-024-01809-9

**Published:** 2024-12-21

**Authors:** Sanjay Kalra, Shahjada Selim, Dina Shrestha, Noel Somasundaram, Syed Abbas Raza, Manash P. Baruah, Saptarshi Bhattacharya, Sharvil Gadve, Ganapathi Bantwal, Rakesh Sahay

**Affiliations:** 1 Bharti Hospital, Kunjpura Road, Karnal, Haryana 132001 India; 2 Department of Endocrinology, Bangabandhu Sheikh Mujib Medical University (BSMMU), Dhaka, Bangladesh; 3 Norvic International Hospital and Medical College, Thapathali, Kathmandu, Nepal; 4 The National Hospital of Sri Lanka, Colombo, Sri Lanka; 5 Shaukat Khanum Cancer Hospital & Research Center, Lahore, Pakistan; 6 Diabetes, Endocrine & Metabolic Disease, Apollo Excelcare Hospitals, Boragaon, Assam India; 7 Endocrinology and Diabetes, Indraprastha Apollo Hospitals, Delhi, India; 8 Excel Endocrine Centre, Diabetes Corner, Kolhapur, Maharashtra India; 9 Department of Endocrinology, St. John’s Medical College & Hospital, Bangalore, Karnataka India; 10 Department of Endocrinology, Osmania Medical College & Osmania General Hospital, Hyderabad, Telangana India

**Keywords:** Graves’ disease, TSH receptor antibodies, TRAbs, South Asia, Best practices, Diagnosis, Prognosis, Monitoring

## Abstract

Graves' disease (GD) is an autoimmune disorder characterized by activation of the TSH receptor by stimulatory autoantibodies (TSH Receptor Antibodies, or TRAbs), leading to unregulated thyroid hormone production. Diagnosis is largely based on the typical clinical picture and laboratory thyroid panel. Establishment of elevated serum levels of TRAbs by competitive binding assay or cell-binding assay has its unique role in diagnosis and management of GD, especially in the differential diagnosis, therapy selection, prognostication, evaluation of thyroid function during pregnancy, peri-conceptional and neonatal thyroid workup, and in certain special situation. Inclusion of TRAbs in GD diagnostic algorithm can improve cost-effectiveness of GD management. The current best practice guidelines were developed to provide evidence-based recommendations in the use of TRABs in GD management for healthcare providers in South Asia. A panel of endocrinologists with minimum 10 years of clinical experience in thyroid disorders reviewed existing literature and their quality, and after deliberation and discussion, developed 21 recommendations surrounding the best practices surrounding the role of TRAbs in GD management.

## Background

Graves’ disease (GD) is the most frequent cause of hyperthyroidism globally, affecting 20–30 out of 100,000 people in iodine-rich regions of the world each year [1]. GD accounts for around 60%-80% of all cases of clinical hyperthyroidism, and is said to affect 1.2% of US adults [2]. While pan-India epidemiological data is lacking, regional estimates suggest that the prevalence of GD in India is also similar, with GD contributing to around 50–80% of the aetiology of hyperthyroidism [1]. It is estimated that GD is prevalent in around 16.7% of the adult population in India [1]. GD affects women more often, with a female: male ratio of around 5–10:1 [1]. While reliable regional data from other countries in the South Asian region are lacking, it can be estimated that GD epidemiology is similar across the subcontinent.

GD is an autoimmune disease associated with an increased production of thyroid hormones (T3 and T4), as well as increase in the size of the thyroid gland, due to the stimulation of thyroid-stimulating hormone (TSH) receptors by autoantibodies [3]. Most patients with GD present with classical thyrotoxicosis symptoms, including sweating, palpitation, heat intolerance, weight loss, tremors, insomnia, anxiety, nervousness, and other features. GD can also present with loss of libido, oligomenorrhoea and amenorrhoea among women. Hyperthyroid clinical picture is easily appreciable on clinical examination with palpable goitre, extrathyroidal manifestations such as Graves ophthalmopathy (GO) and pretibial myxoedema helping to establish the diagnosis [2].

Classical tests useful for establishing thyrotoxicosis include thyroid function tests (low TSH, high T3 and free T4 levels). Tests for establishing the cause of thyrotoxicosis as GD include radioactive iodine (RAI) uptake scan, thyroid ultrasound, and measurement of TSH receptor antibodies (TRAbs). Treatment with thioamide antithyroid drugs (ATD) methimazole (MMI), carbimazole (CBI), and propylthiouracil (PTU) is the most frequently used management option; definitive treatments for non-responders include RAI and thyroidectomy [3].

Different professional bodies have published detailed guidelines pertaining to various facets of management of Graves’ disease, such as the 2016 American Thyroid Association (ATA) guidelines [4] and the 2018 European thyroid association (ETA) guidelines [5] for the diagnosis and management of hyperthyroidism, the 2017 ATA guidelines for the management of peripartum GD [6], the 2022 ETA guidelines [7] and the 2016 Japanese guidelines [8] for the management of paediatric GD. However, apart from a 2011 guidelines on management of Indian patients with thyroid nodules, [9] guidelines specific to management of patients with thyroid disorders in the South Asian region including the Indian subcontinent have not been published.

Studies exploring ethnic differences in autoimmune disorders in general and autoimmune thyroid disorders in particular have unearthed a higher risk and an earlier age of onset of these conditions among South Asians [10, 11]. Thus, the diagnosis, monitoring, and management of autoimmune disorders such as GD are better informed by regional guidelines. Further, considering the improved knowledge in the role of TRAbs in GD and the availability of reliable laboratory tests to measure TRAbs, the need was felt to develop a dedicated recommendation exploring the role of TRAbs in the diagnosis, prognosis, and risk prediction, and management of GD.

Thyroid autoimmunity may result from the formation of autoantibodies that target different thyroid antigens, including thyroglobulin (TG), thyroid peroxidase (TPO), or more importantly, TSH receptor. All antibodies targeting TSH receptor are called by the blanket term TSH Receptor Antibodies (TRAbs). It has been possible to identify three distinct subtypes of TRAbs by means of cell binding assays, based on their overall impact on the TSH receptor: these are Thyroid stimulating antibodies (TSAbs), Thyroid blocking antibodies (TBAbs), and neutral (or ‘cleavage’) antibodies, which stimulate, block, and do not significantly affect, the functioning of the TSH receptor, respectively, as summarised in Table 1. The pathophysiology of autoimmune GD is essentially associated with the persistent and unregulated stimulation of the TSH receptor by TSABs, leading to thyrotoxicosis in the backdrop of low TSH levels [12, 13].

**Table 1:** Types of autoantibodies against TSH receptor

	**Thyroid stimulating antibodies (TSAbs)**	**Thyroid blocking antibodies (TBAbs)**	**Neutral (or ‘cleavage’) antibodies**
**Function**	Stimulate the TSH receptor, mimicking the action of TSH	Block the binding of TSH to the TSH receptor, reducing thyroid stimulation	Compete with TSH for binding to the TSH receptor but do not have a significant stimulatory or inhibitory effect
**Associated condition**	Graves' Disease (Hyperthyroidism)	Some cases of Hypothyroidism	Unclear pathological role, but known to modulate the effects of TSAbs and TBAbs

At the time of their first discovery in 1956, TSAbs were named ‘long-acting thyroid stimulators’ (LATS) [14]. Over the next two decades, the association of TSAbs with GD became stronger through consistent observation of elevated TSABs levels among GD patients. It was also established that the TSAbs bind to the same leucine-rich domain on the A-subunit of the TSH receptor, onto which TSH binds physiologically. The downstream effects of binding of both TSH and TSAbs were also found to be similar. This included generation of cAMP, leading to stimulation of thyroid follicular epithelium proliferation, thyroid growth, release of both thyroid hormones (T3 and T4), and suppression of TSH release from pituitary, which was actually mediated through negative feedback suppression through T3 and T4 [13, 15].

The core clinical feature of GD include palpitations, heat intolerance, and weight loss, due to TSAbs-induced thyrotoxicosis; the diagnostic laboratory findings of GD include typical serum picture of elevated T3 and T4 with suppressed TSH and detectable levels of TRAbs (to be specific, TSAbs) [13]. The TSAbs are in fact considered to be the specific biomarkers for GD [16]. By contrast, the TBAbs bind to TSH receptors and block the action of TSH; these have been associated with hypothyroidism caused by certain forms of autoimmune thyroiditis (such as Hashimoto thyroiditis) [12, 17]. Finally, the neutral antibodies do not appear to affect cAMP generation despite binding to TSH receptors but may induce thyroid cell apoptosis; these antibodies have not yet been linked to any thyroid diseases [12].

Two main types of assays have been developed for the measurement of serum levels of these autoantibodies: the competitive binding assays, and the cell-binding assays, as summarised in Table 2 [13, 18].

**Table 2:** Two main types of thyroid antibody assays

	**Competitive Binding Assays**	**Cell-Binding Assays (bioassays)**
**Principle**	Measure the ability of TRAbs to inhibit TSH receptor ligand binding	Measure the effect of TRAbs on cAMP levels in effector cells, differentiating TSI, TBA, or neutral antibodies
**What is measured**	Concentration of TSH binding inhibiting immunoglobulins (TBIIs) in the patient serum	Concentrations of cAMP levels in the effector cells
**Ligand Used**	1st and 2nd generation: Bovine TSH with porcine/ human TSH receptor; 3rd generation: Biotin-labelled M22 human monoclonal thyroid stimulating antibody with porcine TSH receptor-coated ELISA plates	1st and 2nd generation: Human or porcine thyroid cells; later assays: Chinese hamster cells transfected with human TSH receptor

The competitive binding assays measure the presence of any antibody in the serum that can bind to TSH receptor, irrespective of the downstream effect it produces. In other words, these assays measure the ability of the TRAbs in the serum to inhibit the in-vitro binding of a TSH receptor ligand with the TSH receptor. Since they measure the concentration of TSH binding inhibiting immunoglobulins (TBIIs) in the patient serum, they are also called TBII assays. The first two generations of these assays have used bovine TSH as the TSH receptor ligand, and porcine (1st and 2nd generation) or human (2nd generation) TSH receptor to measure serum TRABs, but the low specificity of detection was a concern [13].

The third-generation assay, developed in 2004, uses the biotin-labelled M22 human monoclonal thyroid stimulating antibody as the ligand, and porcine TSH receptor-coated ELISA (enzyme-linked immunosorbent assay) plates [19]. The automated version of this assay that was developed in 2007 replaced the unit of measurement from percentage of inhibition to IU/L, and the cut-off point of 1.75 IU/L in this assay was associated with a 99% sensitivity and specificity for the diagnosis of GD [20]. The upper limits of TRAbs among healthy individuals and among patients with non-GD thyroid dysfunction have been determined as 1.22 IU/L and 1.58 IU/L respectively [20]. The optimal cut-off levels for TRAbs, however, depends on several factors including methodology of assay and calibration standards, and manufacturer-recommended cut-offs are required to be used as the benchmark for the diagnosis of GD [21]. For similar reasons, third generation assays are not directly comparable with each other [22]. For instance, the Elecsys assay (Roche Diagnostics International Ltd, Basel, Switzerland) that uses electrochemiluminiscent immunoassay (ECLIA) in a COBAS 6000 auto-analyzer has a cut-off of 1.75 IU/L for GD diagnosis; however, when interpreting TRAbs results from the EliA assay (Phadia A.B, Thermo Fisher Scientific, Uppsala, Sweden) that uses fluorescence enzyme immunoassay (FEIA) in the Phadia 250 auto-analyzer, a value of < 2.9 IU/L is negative for GD, > 3.3 IU/L is positive for GD, and 2.9–3.3 IU/L are categorized as grey zone, and the results are said to be ‘intermediate’ [21].

The cell-binding assays on the other hand measure the effect of TRAbs binding to the TSH receptor, in terms of alterations in the concentrations of cAMP levels in the effector cells. As a result, these assays have the ability to differentiate the exact type of TRAbs into either TSAbs, TBAbs, or neutral antibodies [12, 13]. Since these biological assays can specifically identify thyroid stimulating immunoglobulins (TSIs), these assays are also called TSI bioassays. The effector cells were human or porcine thyroid cells in the 1st generation assays, and Chinese hamster cells transfected with human TSH receptor in the later assays. The detection of cAMP concentration in the effector cells is achieved through radioimmune assay (1st and 2nd generations) or using a luciferase reporter (3rd generation). The 3rd generation assays are also automated, and have a higher specificity than the earlier generations [13]. The bioassays can also provide information pertaining to the potency and functional activity of the autoantibodies [16].

The TSI bioassays provide valuable information about the type of TRAbs which the TBII assays cannot provide. However, while the TBII assays are relatively cheap and easy to perform, the TSI bioassays are generally more expensive, technically complex and time-consuming [23]. A comparative specificity of the automated versions of both these assays in diagnosis of various thyroid disorders is summarised in Table 3 [13].

**Table 3:** Percentage of individuals with different conditions, and healthy individuals, showing positive antibody titre by different assays [Adapted from Kotwal and Stan, 2018[13]]

Type of individuals	3rd generation automated Competition-based TBII assay	3rd generation automated TSI bioassay
Newly diagnosed Graves’ Disease	99%	98.9%
Chronic autoimmune (Hashimoto’s) thyroiditis	1–2%	7.8%
Painless/ silent thyroiditis	1%	2.3%
Thyroid cancer	11%	NA
Healthy individuals	0.6%	0%

The advantages of measurement of TRAbs are summarised in Table 4.

**Table 4:** Advantages of measuring TRAbs

Measurement of TRAbs has a definitive role in various thyroid disorders:
• Differential diagnosis of thyrotoxicosis
• Diagnosis, therapy selection, and prognosis of GD
• Evaluation of thyroid function during pregnancy
• Peri-conceptional and neonatal thyroid workup
• In special situations such as of Graves’ dermopathy (pretibial myxoedema) and Graves’ ophthalmopathy in clinically euthyroid patients, and thyrotoxicosis complicating immune reconstitution syndrome [13, 23]
Based on an evidence-based economic model, the inclusion of TRAbs in GD diagnostic algorithm was found to improve the time to diagnosis by 46%, and reduce the cost of diagnosis by 47% [24], and the definitive diagnosis established by these tests will obviate the need for additional tests [25]

With this background, and also noting the dearth of guidelines specific to the use of TRAbs catering to the diagnosis and management of patients with GD and related conditions in the South Asian region, we aim to develop these best practice guidelines.

## Methods

The development of this document was conceived by a panel of South Asian endocrinologists with minimum 10 years of experience managing patients with thyroid disorders. The members consenting to be a part of the expert panel communicated with each other informally through virtual social groups almost continuously, and formally through minuted virtual meetings three times: once before the drafting the best practice points, once to discuss, deliberate, vote, and finalise the best practice points, and once to finalise the manuscript.

The best practice points were based on a targeted literature search in PubMed directed by headings that were decided by the expert panel. Each best practice point was supported by evidence from literature, and was accompanied by the quality of evidence and strength of recommendations. The GRADE (Grading of Recommendations, Assessment, Development, and Evaluation) system was used to first categorise the quality of evidence into high, moderate, low, and very low quality, and based on this, the strength of recommendation was judged to be either “strong” and “weak/ conditional/ discretionary” [26].

After the best practice points were drafted, each point was individually discussed by the expert panel; following a detailed deliberation and review of evidence, a voting was held by the members. The outcome of this voting in terms of consensus achieved was displayed along with the quality of evidence and strength of evidence. This enables the reader to take informed decision pertaining to the adoption of the guidance in routine practice.

## Best practice recommendations

### Role in diagnosis

**Table:** 

R1. The measurement of TRAbs is a sensitive and specific tool for differential diagnosis of thyrotoxicosis R2. The measurement of TRAbs is a sensitive and specific tool for accurate and rapid diagnosis of Graves’ disease	
**Quality of evidence**	**Strength of Recommendation**
High/ Moderate	Strong

Serum TSH measurement has the highest sensitivity and specificity when used as an initial screening test for thyrotoxicosis, and the diagnostic accuracy improves when serum TSH, free T4, and total T3 are assessed together at initial evaluation [27]. If the aetiology of thyrotoxicosis cannot be reasonably established by clinical presentation and basic biochemical evaluation, diagnostic testing should be performed. Available modalities include radioactive iodine uptake (RAIU), thyroid blood flow evaluation through ultrasound scan, and measurement of TRAbs. RAIU cannot distinguish between thyrotoxicosis due to painless, subacute, or postpartum thyroiditis, or thyrotoxicosis due to recent excess iodine or thyroid hormone intake. Further, RAIU results are affected by factors such as recent iodine contrast media exposure and ingestion of diet rich in iodine, and also require dedicated setup and technical expertise.

Though thyroid ultrasound scan can reliably identify thyroid overactivity by measuring increased thyroid blood flow, it requires technical expertise. By contrast, estimation of TRAbs is cost-effective by 47%, and improves time to diagnosis by 46%, compared to non-TRAbs modes of diagnosis [24]. Being laboratory-based, TRAbs estimation does not require a dedicated setup or special technical expertise. Further, since TRAbs are considered the specific biomarkers for GD [16], a positive TRAb result confirms the diagnosis of GD which is the most frequent cause of thyrotoxicosis. This is cost-effective as it obviates the need to undergo RAIU or thyroid ultrasound examinations for a significant proportion of thyrotoxicosis patients. The 3rd generation TBII assays have a sensitivity of 97.4% and specificity of 99.2% for GD diagnosis [28]. Thus, depending on the availability, we recommend that TRAbs can be used as sensitive and specific tools for differential diagnosis of thyrotoxicosis, and also for the rapid and accurate diagnosis of GD in all patients with clinical and biochemical thyrotoxicosis.

### Role in management planning

#### Risk stratification/ prognostication

**Table:** 

R3. Baseline levels of TRAbs, along with other clinical indicators, can help in predicting treatment response to therapy in Graves’ Disease R4. Baseline levels of TRAbs can help in predicting prognosis and recurrence of Graves’ Disease, especially in young individuals	
**Quality of evidence**	**Strength of Recommendation**
Moderate	Strong

After 24 months of therapy with methimazole (MMI), 63.3% of patients with baseline TRAbs values of < 10 IU/L were observed to attain remission, and the mean time of entering remission was 16.4 months; the corresponding values for patients with baseline TRAbs > 10 IU/L were 39.4% and 21.5 months respectively (*p* < 0.05 in both cases). The sensitivity and specificity for baseline TRAbs levels of 10 IU/L to predict remission were 57% and 74% respectively [29]. In another study, GD patients with baseline TRAbs value ≥ 46.5 IU/L failed to achieve remission, with a sensitivity and specificity of 52% and 78% respectively [30]. Higher baseline TRAbs levels were associated with greater risk of GD relapse, (HR 1.05, 95% CI 1.02–1.08, *p* = 0.001) independent of age, sex, race, smoking status, and TPOAb levels; the association was more apparent among younger patients (18–41 years, HR 1.13, 95% CI 1.04–1.23, *p* = 0.005; 42–56 years, HR 1.05, 95% CI 1.01–1.09, *p* = 0.01) but was not significant in patients aged ≥ 57 years (HR0.99, 95% CI 0.93–1.05, *p* = 0.7) [31]. Higher baseline TRAbs levels were also associated with poorer prognosis after 12–18 months of therapy, irrespective of whether the patients received antithyroid drugs, radioiodine therapy, or surgical therapy [32].

A scoring system was constructed and validated for predicting the risk of recurrent GD after 18 months of antithyroid drug (ATD) therapy using four baseline variables: age, serum fT4, TRAbs, and goitre size; the resultant GREAT (Graves’ Recurrent Events After Therapy) score of 0–1, 2–3, and 4–6 were associated with a recurrence risk of 16%, 44%, and 68% respectively [33, 34]. Thus, by considering baseline TRAbs in addition to clinical variables such as age, gender, thyroid volume, and severity of hyperthyroidism, it is possible to identify and stratify patients into categories with low or high risk of remission following treatment, as well as predict the prognosis and recurrence of GD [35].

As an extension to this observation, recording of baseline TRAbs will also help in counselling patients about the possible duration of therapy, importance of medication adherence, and treatment outcomes, thereby allowing an informed decision-making on the part of the patient about the most appropriate format of thyrotoxicosis therapy.

#### Treatment monitoring and follow up

**Table:** 

R5. Measurement of serum TRAb levels after 12–18 months of ATD therapy should inform decision regarding further management of Graves’ disease R6. Antithyroid therapy with either ATDs or RAI or thyroidectomy should be offered to patients in whom serum TRAbs levels are persistently high after 12–18 months of therapy with ATDs	
**Quality of evidence**	**Strength of Recommendation**
High/ Moderate	Strong
**R7.** Evidence is insufficient to recommend regular TRAb level estimation in addition to fT4 and TSH levels for monitoring antithyroid drug (ATD) treatment response in Graves’ disease	
**Quality of evidence**	**Strength of Recommendation**
Moderate/ Low	Conditional

Treatment of thyrotoxicosis arising from GD hinges on the principles of reducing T3 and T4 synthesis from the thyroid gland, by using modalities such as the antithyroid drugs (ATDs) methimazole (MMI), carbimazole (CBZ) and propylthiouracil (PTU), or thyroid ablation using radioactive iodine (RAI) or thyroidectomy. ATDs are the first-line therapy for GD in most patients, especially among younger patients, and also for achieving euthyroid status prior to RAI or thyroidectomy [5]. Upon initiation of ATD therapy, secondary to their suppression of T3 and T4 production by the thyroid, there is a fall in the serum levels of T3, T4, and consequently of TSH as well. Optimal ATD dose is achieved by monitoring fT4 and TSH levels usually every 3–4 weeks; in fact, a large proportion of patients become clinically and biochemically euthyroid within 3–4 weeks of treatment initiation.

Remission in GD is usually defined as normal serum levels of T3, fT4, and TSH after 1 year of treatment discontinuation. Remission rates in GD vary significantly from 30–70% in various studies [36]. Lower remission rates are seen among children, men, smokers, patients with large goitres, and patients with more active disease [37]. The optimal duration of maintenance therapy with ATDs is 12–18 months. Remission is not influenced by the duration of therapy, and prolonged therapy with ATD is more often associated with adverse effects [5, 38]. At the end of 18 months of ATD therapy, TRAb assessment will help define further course of action. Patients with low or undetectable TRAbs levels have a high probability of permanent remission: GD may relapse in around 20–30% of such patients after stopping ATD therapy. On the other hand, patients with persistently elevated TRAbs levels are unlikely to be in remission and require further therapy, and GD may relapse in around 80–100% of such patients after stopping ATD therapy [4]. Patients with elevated TRAbs levels after 18 months of ATD can be counselled to continue ATD therapy and repeat TRAbs measurement after another 12–18 months. Alternatively, definitive treatments such as RAI or thyroidectomy may be offered to these patients.

ATD treatment monitoring may be done by clinical examination and measuring serum levels of T3, fT4 and TSH at regular intervals, which can range from once every 2–6 months [39]. Lack of normalization of TRAbs level during therapy was associated with ineffective treatment in a study; [32] however, in this study TRAbs levels were estimated at baseline and after 12 or 18 months of therapy. Evidence is insufficient to recommend measurement of serum TRAbs levels in shorter intervals for monitoring of ATD treatment efficacy.

### Role in specific situations

#### Preconception

**Table:** 

R8. TRAbs levels among female GD patients who are planning conception guide treatment selection R9. Definitive therapy (radioactive iodine therapy or thyroid surgery) may be recommended over ATD if TRAbs levels are very high	
**Quality of evidence**	**Strength of Recommendation**
High/ Moderate	Strong

Hyperthyroidism is estimated to occur in around 1–2 cases per 1000 pregnancies [40]. Uncorrected maternal hyperthyroidism, especially during the early gestation period, is associated with foetal, neonatal, and maternal adverse outcomes. Overenthusiastic usage of ATDs for GD management during pregnancy can also lead to unwanted foetal outcomes, as a result of side effect to ATDs, or due to the induced hypothyroidism. Finally, maternal TRAbs can also cross the placenta at or after 20 weeks of pregnancy and induce fatal thyroid dysfunction, which may result in either hypothyroidism or hyperthyroidism in the foetus depending on the nature of the TRAbs [6, 41]. Considering all these reasons, a meticulous preconception strategy is essential in patients with GD who are planning conception, with an aim to reduce the risk of maternal hyperthyroidism and ATD usage from negatively impacting the pregnancy outcomes [41].

Women with GD must be counselled about the risks of foetal harms due to uncontrolled hyperthyroidism, and must be advised to postpone conception until they have achieved a stable euthyroid state. All three treatment options (ATD, RAI, and surgery) must be offered to these women. Women in whom the risk of GD relapse is high are generally considered better candidates for definitive therapy with RAI or surgery [42]. Identification of such women can be reliably done by measuring TRAbs levels, in addition to clinical and biochemical features [43]. Women with very high TRAb levels may be best suited for thyroidectomy [44]. For women opting for RAI, it is essential to delay pregnancy by a further 6 months, to avoid foetal harm resulting from radioiodine exposure [6]. Also, it is essential to perform a pregnancy test to all women prior to RAI therapy. Finally, it must be noted that TRAbs levels may stay elevated after RAI and surgery for several months up to a year [45, 46].

#### Pregnancy

**Table:** 

R10. Low maternal TRAbs levels, in addition to other indicators such as disease history, goitre size, duration of therapy, and clinical indicators, can be considered while deciding to withhold ATD therapy among pregnant GD women who achieve clinical and biochemical euthyroid state	
**Quality of evidence**	**Strength of Recommendation**
Low	Conditional
**R11.** All pregnant women with current GD irrespective of treatment status, euthyroid pregnant women with past GD, and women with history of delivering an infant with neonatal hypothyroidism, must undergo TRAbs estimation to evaluate the risk of foetal and neonatal thyroid dysfunction, at first contact with antenatal care team **R12.** TRABs helps in the differential diagnosis of GD from gestational thyrotoxicosis **R13.** If TRAbs levels are elevated in early pregnancy, the levels must be reassessed at weeks 18–22, and again at weeks 30–34, to evaluate the need for monitoring the neonate for thyroid dysfunction **R14.** High maternal serum TRAbs level in women with GD during 2nd trimester is prognostic for development of neonatal hyperthyroidism **R15.** Maternal TRAbs level estimation should guide ATD dose modification when there is evidence of foetal goitre in women with GD on ATD therapy	
**Quality of evidence**	**Strength of Recommendation**
High	Strong

TRAbs has an essential role in management of GD during pregnancy. ATDs are the mainstay of management of GD during pregnancy, and the starting dose depends on degree of thyrotoxicosis and severity of symptoms [6, 47]. If women with GD who are clinically and biochemically euthyroid while on low-dose ATD (≤ 5–10 mg/d MMI, ≤ 100–200 mg/d PTU) conceives, depending upon other factors including disease history, goitre size, duration of therapy, TRAbs levels, and clinical indicators, a decision to withhold ATD may be taken, for protecting the foetus from the potential teratogenic effects of the ATDs [6]. It must be remembered that relapse risk of GD among euthyroid women on ATD after withdrawal of therapy is high in patients who have received therapy for < 6 months, who require > 5–10 mg/d MMI to stay euthyroid, have high levels of TRAbs, have large goitre, and active orbitopathy [43].

By the 3rd trimester of pregnancy, there is a general decrease in autoimmunity, as evidenced by a decrease in maternal TRAbs level; this translates to a general improvement in GD. As a result, in 20–30% of GD patients it is possible to attempt an ATD withdrawal. Disappearance of TRAbs in late pregnancy generally indicates that the ATD withdrawal has a high probability of success [6, 48].

Recently, TRAbs has also been proposed as a novel risk indicator for adverse pregnancy outcomes, and an optimal cut-off value of 3.53 IU/L was found to be associated an increased risk of pregnancy loss, with a sensitivity and specificity of 83.5% and 85.3%, respectively [49].

TRAbs estimation among pregnant GD women also has a strong role in predicting the development of neonatal thyroid dysfunction. For this purpose, it is recommended that all women with GD who have conceived, irrespective of treatment status, must undergo TRAbs testing. Also, TRAbs levels must be estimated among euthyroid women who have previously undergone RAI therapy or surgery for GD [6, 50], and among women who have previously delivered an infant with neonatal hypothyroidism [23]. In a 2014 study, it was found that among the neonates born to mothers with GD, neonatal hyperthyroidism was found only among those neonates whose mothers had circulating TRAbs [51]. Maternal TRAb levels of > 5 IU/L during the 2nd trimester of pregnancy were associated with neonatal hyperthyroidism with sensitivity and specificity rates of 100% and 43% respectively [52]. In another study, maternal TRAb values of ≥ 5.9 IU/L were predictive of neonatal thyroid hypertrophy (accompanied by either thyrotoxicosis or hypothyroidism), with sensitivity and specificity rates of 100% and 82% respectively [53].

Foetuses of pregnant women with GD on ATD treatment may develop goitre through two mechanisms, both of which require opposite modalities of treatment. For goitres developing due to maternal ATD therapy causing foetal thyroid suppression, a reduction in maternal ATD dose is recommended, and for goitres developing due to foetal thyroid stimulation by maternal TRAbs crossing the placenta, an increase in maternal ATD dose is recommended. The estimation of maternal TRAbs level is crucial for this treatment decision [50].

Based on these observations, we also recommend that if foetal goitre is documented, then maternal TRAbs level estimation should be done to determine further course of action.

#### Neonatal thyroid dysfunction

**Table:** 

R16. Elevated TRAbs levels in newborn serum or cord blood, along with elevated thyroid hormone levels, should inform the initiation of ATD therapy in the neonate	
**Quality of evidence**	**Strength of Recommendation**
High	Strong

Routine newborn screening for neonatal hypothyroidism is done in many developed countries. TRAbs estimation in newborn also has utility in certain specific conditions. If serum maternal TRAbs are detectable at the end of gestation, neonatal thyroid dysfunction can be suspected due to the transplacental effect of the same. In such cases, it is recommended that thyroid function tests be performed every 2 days in the first week of life [50]. Serum TRAbs levels in the newborn ≥ 6.8 IU/L, measured in either peripheral blood sample before day 5 of birth, are predictive of neonatal thyroid dysfunction with sensitivity and specificity rates of 100% and 94% respectively [53]. High newborn TRAbs levels, accompanied by elevated thyroid hormone levels in the first week of life, should lead to initiation of ATD in the neonate for preventing clinical hyperthyroidism [50].

#### Postpartum

**Table:** 

R17. Postpartum TRAbs measurement may help distinguish postpartum thyroiditis presenting as clinical hyperthyroidism from GD	
**Quality of evidence**	**Strength of Recommendation**
Low/ Moderate	Conditional

Postpartum thyroiditis (PPT) is associated with autoantibodies to TPO, and the destructive autoimmune process may present clinically as transient hyperthyroidism, transient hypothyroidism, or hyperthyroidism followed by hypothyroidism and recovery [54]. When presenting as hyperthyroidism, measurement of TRAbs can help distinguish PPT from GD [55]; this distinction is essential because ATDs which are the mainstay treatment for GD, are ineffective in the treatment of PPT. Postpartum elevation of TRAbs (specially TSAbs) can also predict recurrence of GD [55].

#### Paediatric GD

**Table:** 

R18. Low TRAbs level, along with small thyroid gland size, are associated with higher possibility of remission in paediatric GD R19. Stopping of ATD therapy in paediatric GD patients should be based on TRAbs level after at least 3 years of ATD therapy, and treatment should be continued for 5 years or potentially longer for patients with high TRAbs levels	
**Quality of evidence**	**Strength of Recommendation**
Moderate	Strong

GD is the most common cause of hyperthyroidism in children, and children aged ≥ 10 years constitute around 80% of paediatric GD cases [56]. Baseline TRAbs levels were found to correlate significantly with severity of hyperthyroidism [57]. Another study found serum TRAbs level normalization, along with an absence of goitre and lower median TRAbs levels, to be associated with better treatment outcomes [58]. In paediatric GD patients, ATD therapy is normally administered for 3 years; the subsequent treatment decision depends on the dose of ATD resulting in euthyroid state, and the likelihood of long-term remission of GD. Factors known to be associated with higher likelihood of remission of paediatric GD include older age, female sex, smaller goitre size, mild biochemical disease at diagnosis, longer duration of ATD treatment, and lower TRAbs titres [7]. Most patients responding to ATD show a median decline in TRAbs by 90% within 3 years of treatment [58, 59]; patients with high titres of TRAbs generally have a low probability of remission, and ATD therapy should be continued in such patients for 5 years or even longer. Additionally, documenting high TRAbs levels can also help in counselling regarding various treatment options [56].

#### Diagnosis of Thyroid-associated orbitopathy (TAO)

**Table:** 

R20. Baseline TRAbs value can provide prognosis about the risk of patients developing severe TAO, but evidence is insufficient to recommend using TRAbs level for treatment decisions in TAO	
**Quality of evidence**	**Strength of Recommendation**
Low/ Moderate	Conditional

Thyroid-associated orbitopathy (TAO), also called Graves Orbitopathy (GO), affects at least 30–50% of GD patients. In the presence of other clinical and laboratory features of GD, the diagnosis of TAO is quite straightforward. However, TRAbs estimation is required for the diagnosis of TAO in certain special situations, such as when TAO is seen in the absence of other clinical features of GD, and when TAO is rarely seen in hypothyroid patients [23]. The main role of TRAbs in TAO is in prognostication: patients with higher baseline TRAbs value have a higher risk of severe TAO, and values of TRAbs ≥ 10.67 IU/l are predictive of severe course of TAO, with sensitivity and specificity rates of 66.7% and 84.9% respectively [60]. Another study concluded that TRAbs levels > 8.8 IU/l are associated with high risk of progression of TAO [61]. Evidence is insufficient to recommend using TRAbs levels to determine the need for immunomodulation in TAO therapy [13]. Finally, GD patients undergoing RAI therapy are at an increased risk of developing TAO, and a surge in TRAbs after RAI therapy was found to not have a prognostic significance for predicting TAO occurrence post therapy [62].

#### Dermatopathy

**Table:** 

R21. Evidence is not sufficient to recommend TRAbs estimation for diagnosis, prognosis, or management of Graves’ dermopathy	
**Quality of evidence**	**Strength of Recommendation**
Low/ Moderate	Conditional

Serum TRAb levels have been positively correlated with the course and severity of Graves’ dermopathy (also called pretibial myxoedema) [63]; however, evidence is not sufficient to recommend routine estimation of TRAbs for diagnosis, prognosis, or management of this condition.

All the best practice recommendations are summarised in Table 5.

**Table 5:** Summary of best practice recommendations for the role of TRAbs in the diagnosis, prognostication, prediction and monitoring of Graves’ disease

Recommendation	Quality of evidence	Strength of Recommendation
**R1.** The measurement of TRAbs is a sensitive and specific tool for differential diagnosis of thyrotoxicosis	High/ Moderate	Strong
**R2.** The measurement of TRAbs is a sensitive and specific tool for accurate and rapid diagnosis of Graves’ disease	High/ Moderate	Strong
**R3.** Baseline levels of TRAbs, along with other clinical indicators, can help in predicting treatment response to therapy in Graves’ Disease	Moderate	Strong
**R4.** Baseline levels of TRAbs can help in predicting prognosis and recurrence of Graves’ Disease, especially in young individuals	Moderate	Strong
**R5.** Measurement of serum TRAb levels after 12–18 months of ATD therapy should inform decision regarding further management of Graves’ disease	High/ Moderate	Strong
**R6.** Antithyroid therapy with either ATDs or RAI or thyroidectomy should be offered to patients in whom serum TRAbs levels are persistently high after 12–18 months of therapy with ATDs	High/ Moderate	Strong
**R7.** Evidence is insufficient to recommend regular TRAb level estimation in addition to fT4 and TSH levels for monitoring antithyroid drug (ATD) treatment response in Graves’ disease	Moderate/ Low	Conditional
**R8.** TRAbs levels among female GD patients who are planning conception guide treatment selection	High/ Moderate	Strong
**R9.** Definitive therapy (radioactive iodine therapy or thyroid surgery) may be recommended over ATD if TRAbs levels are very high	High/ Moderate	Strong
**R10.** Low maternal TRAbs levels, in addition to other indicators such as disease history, goitre size, duration of therapy, and clinical indicators, can be considered while deciding to withhold ATD therapy among pregnant GD women who achieve clinical and biochemical euthyroid state	Low	Conditional
**R11.** All pregnant women with current GD irrespective of treatment status, euthyroid pregnant women with past GD, and women with history of delivering an infant with neonatal hypothyroidism, must undergo TRAbs estimation to evaluate the risk of foetal and neonatal thyroid dysfunction, first contact with antenatal care team	High	Strong
**R12.** TRABs helps in the differential diagnosis of GD from gestational thyrotoxicosis	High	Strong
**R13.** If TRAbs levels are elevated in early pregnancy, the levels must be reassessed at weeks 18–22, and again at weeks 30–34, to evaluate the need for monitoring the neonate for thyroid dysfunction	High	Strong
**R14.** High maternal serum TRAbs level in women with GD during 2nd trimester is prognostic for development of neonatal hyperthyroidism	High	Strong
**R15.** Maternal TRAbs level estimation should guide ATD dose modification when there is evidence of foetal goitre in women with GD on ATD therapy	High	Strong
**R16.** Elevated TRAbs levels in newborn serum or cord blood, along with elevated thyroid hormone levels, should inform the initiation of ATD therapy in the neonate	High	Strong
**R17.** Postpartum TRAbs measurement may help distinguish postpartum thyroiditis presenting as clinical hyperthyroidism from GD	Low/ Moderate	Conditional
**R18.** Low TRAbs level, along with small thyroid gland size, are associated with higher possibility of remission in paediatric GD	Moderate	Strong
**R19.** Stopping of ATD therapy in paediatric GD patients should be based on TRAbs level after at least 3 years of ATD therapy, and treatment should be continued for 5 years or potentially longer for patients with high TRAbs levels	Moderate	Strong
**R20.** Baseline TRAbs value can provide prognosis about the risk of patients developing severe TAO, but evidence is insufficient to recommend using TRAbs level for treatment decisions in TAO	Low/ Moderate	Conditional
**R21.** Evidence is not sufficient to recommend TRAbs estimation for diagnosis, prognosis, or management of Graves’ dermopathy	Low/ Moderate	Conditional

**Fig. 1:**
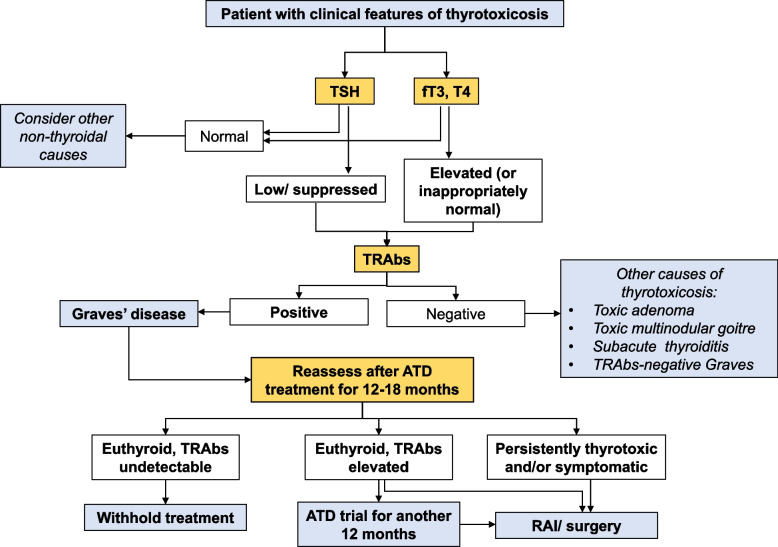
Recommended algorithm for laboratory management of Graves’ Disease in non-pregnant adult patients

A suggested algorithm for the laboratory management of GD in non-pregnant adult patients that incorporates the usage of TRAbs that incorporates the relevant points of this best practice paper is provided in Fig. 1.

## Relevance of TRAbs in the South Asian setting

Being the specific marker of GD, it is no surprise that TRAbs has a key role in the diagnosis, prognostication, risk stratification, and management of GD in adults, pregnant women, and in neonates. TRAbs has been regularly used in the developed countries for GD management, as evidenced by various guidelines recommending TRAbs use in various situations [4–6]. The 3rd generation TRAbs assays that are approved and currently available for use have a high specificity for diagnosis of GD. It is also now known that routine use of TRAbs assay as per approved indications and algorithm can be cost-effective despite the high upfront cost of the test, and reduce the time to diagnosis [24]. By establishing the definitive diagnosis of GD, TRAbs assay also obviates the need for alternative diagnostic modalities for GD such as USG and RAI uptake, thereby improving patient satisfaction [25]. The rational use of TRABs will help in the diagnosis, monitoring, and management of persons with GD.

## Study limitations

These recommendations are the result of expert opinion from a group of endocrinologists, and need validation from a larger cohort of specialists. As of the time of this publication, the availability of TRAbs in the South Asian region is limited, and not as widespread as other diagnostic modalities; efforts should be made for widespread availability and usage of the TRAbs assay. There is also a need for a formal cost-effectiveness estimation of TRAbs compared to other diagnostic modalities for GD.

## Conclusions

TRAbs has a definitive role in the diagnosis, prognostication, prediction, and monitoring of GD in adult patients. It also has a role in the management of GD in preconception counselling, GD in pregnancy, neonatal thyroid dysfunction, differential diagnosis of postpartum thyroiditis, and Graves ophthalmopathy. Rational usage of TRAbs assay can be cost-effective and also reduce the time required for diagnosis, thereby improving patient satisfaction. This is relevant in the South Asian setting.

## Data Availability

No datasets were generated or analysed during the current study.
